# Recombinant Uncarboxylated Osteocalcin *Per Se* Enhances Mouse Skeletal Muscle Glucose Uptake in both Extensor Digitorum Longus and Soleus Muscles

**DOI:** 10.3389/fendo.2017.00330

**Published:** 2017-11-22

**Authors:** Xuzhu Lin, Lewan Parker, Emma Mclennan, Xinmei Zhang, Alan Hayes, Glenn McConell, Tara C. Brennan-Speranza, Itamar Levinger

**Affiliations:** ^1^Institute of Sport, Exercise and Active Living (ISEAL), Victoria University, Melbourne, VIC, Australia; ^2^School of Exercise and Nutrition Sciences, Institute for Physical Activity and Nutrition (IPAN), Deakin University, Melbourne, VIC, Australia; ^3^College of Health and Biomedicine, Victoria University, Geelong, VIC, Australia; ^4^Australian Institute for Musculoskeletal Science, Western Health, Melbourne, VIC, Australia; ^5^Department of Physiology, Bosch Institute for Medical Research, University of Sydney, Sydney, NSW, Australia

**Keywords:** undercarboxylated osteocalcin, skeletal muscle, glucose uptake, extracellular signal-regulated kinase, adenosine monophosphate-activated protein kinase, mechanistic target of rapamycin complex 2-AKT-AS160 signaling cascade

## Abstract

Emerging evidence suggests that undercarboxylated osteocalcin (ucOC) improves muscle glucose uptake in rodents. However, whether ucOC can directly increase glucose uptake in both glycolytic and oxidative muscles and the possible mechanisms of action still need further exploration. We tested the hypothesis that ucOC *per se* stimulates muscle glucose uptake *via* extracellular signal-regulated kinase (ERK), adenosine monophosphate-activated protein kinase (AMPK), and/or the mechanistic target of rapamycin complex 2 (mTORC2)-protein kinase B (AKT)-AKT substrate of 160 kDa (AS160) signaling cascade. Extensor digitorum longus (EDL) and soleus muscles from male C57BL/6 mice were isolated, divided into halves, and then incubated with ucOC with or without the pretreatment of ERK inhibitor U0126. ucOC increased muscle glucose uptake in both EDL and soleus. It also enhanced phosphorylation of ERK2 (Thr202/Tyr204) and AS160 (Thr642) in both muscle types and increased mTOR phosphorylation (Ser2481) in EDL only. ucOC had no significant effect on the phosphorylation of AMPKα (Thr172). The inhibition of ucOC-induced ERK phosphorylation had limited effect on ucOC-stimulated glucose uptake and AS160 phosphorylation in both muscle types, but appeared to inhibit the elevation in AKT phosphorylation only in EDL. Taken together, ucOC at the physiological range directly increased glucose uptake in both EDL and soleus muscles in mouse. The molecular mechanisms behind this ucOC effect on muscle glucose uptake seem to be muscle type-specific, involving enhanced phosphorylation of AS160 but limitedly modulated by ERK phosphorylation. Our study suggests that, since ucOC increases muscle glucose uptake without insulin, it could be considered as a potential agent to improve muscle glucose uptake in insulin resistant conditions.

## Introduction

The skeleton is an endocrine organ that has been shown, at least in mice, to modulate glucose metabolism (1–3). One bone-specific hormone that plays a role in this energy regulation is osteocalcin (OC) (4, 5). Undercarboxylated osteocalcin (ucOC), the biologically active form of OC, regulates glucose metabolism by targeting the pancreas and perhaps several insulin-sensitive organs, including skeletal muscle (6–8). The effect of ucOC on skeletal muscle may have important clinical implications for whole-body glycemic control as it is the major site for glucose disposal and storage (9, 10). It has been reported that ucOC increases insulin sensitivity in rodent skeletal muscle (11–15). Recent evidence also suggests that ucOC may enhance muscle glucose uptake in the absence of insulin. For example, it has been shown that 10 ng mL^−1^ ucOC increases glucose uptake in C2C12 myotubes, and to a lesser extent in *ex vivo* soleus muscle which mainly relies on oxidative metabolism for energy production, but not in *ex vivo* extensor digitorum longus (EDL) muscle, which largely utilize glycolytic metabolism as the energy source (15). Similarly, in our previous study, we did not observe any effect of ucOC on glucose uptake of non-contracted EDL *ex vivo* (14). However, since GPRC6A, the presumable receptor for ucOC, is expressed in both EDL and soleus (14, 15), the regulation on muscle glucose uptake by ucOC in both muscle types is still possible. We hypothesize that the limited direct effects of ucOC that was previously observed on EDL were likely due to the inadequateness of ucOC to access the internal area of intact muscle *in vitro*. Therefore, it is possible that a methodological limitation affected the results and improved techniques such as the application of muscle strips, which was previously performed by Cartee et al. (16) and others (17, 18), need to be introduced.

Furthermore, the potential mechanisms behind ucOC *per se* effect on skeletal muscle glucose uptake are still largely unknown. Our previous report exhibited enhanced insulin-stimulated glucose uptake and AS160 phosphorylation at Thr642 by ucOC treatment in EDL muscle post *ex vivo* contraction (14). Insulin-induced phosphorylation of AS160, and subsequent increases in glucose uptake, requires fully activated AKT *via* the activation of mechanistic target of rapamycin complex 2 (mTORC2), which can be indicated by the phosphorylation of AKT at Ser473 and the phosphorylation of mTOR at Ser 2481 (19–22). The mTORC2-AKT-AS160 signaling cascade can be stimulated not only by insulin but also other growth factors and stimuli (23–25). Recent findings indicate that ucOC may also be able to trigger this signaling pathway. In vascular smooth muscle cells, the phosphorylation of AKT was enhanced by the treatment of purified bovine OC (26). Furthermore, the phosphorylation of AKT at Ser473 was elevated following OC treatment in descending thoracic aortic strips of ApoE-KO mice (27). In addition, the phosphorylation of AKT (Ser473) was increased in C2C12 myotubes with ucOC exposure during cell differentiation (13). Nevertheless, the upstream pathway/s that result in the phosphorylation of AKT and AS160 by ucOC are still unclear. It is possible that two previously identified downstream targets of ucOC, extracellular signal-regulated kinase (ERK) and adenosine monophosphate-activated protein kinase (AMPK), may be involved (28, 29). Indeed, in atrophic rat muscles, lower serum ucOC levels were associated with lower phosphorylation levels of ERK (Thr202/Tyr204) and AMPK (Thr172), and the phosphorylation levels of ERK positively correlated with the phosphorylation levels of AKT (S473) in EDL muscle (30). In C2C12 myotubes, ucOC-stimulated ERK phosphorylation (Thr202/Tyr204) likely contributed to the increase of AKT phosphorylation at Ser473 (13). Furthermore, exercise-induced p-AMPK (Thr172) was augmented by ucOC injection in mice tibialis muscle, which could be responsible for ucOC-enhanced exercise-stimulated muscle glucose uptake (15).

Therefore, the aims of this study were to (a) test the hypothesis that physiological levels of ucOC *per se* increases glucose uptake in both EDL and soleus muscles and (b) explore the mechanisms underlying the effects of ucOC on muscle glucose uptake.

## Materials and Methods

### Animals

Eight-week-old male C57BL/6J mice (*N* = 55) were purchased from Animal Resources Centre (WA, Australia). All mice were group housed with a 12-h light/12-h dark cycle and fed standard laboratory chow [Specialty Feeds mouse food cubes (Glen Forrest, WA, Australia) containing 20% protein, 4.8% fat, and the rest carbohydrate and fiber] and water *ad libitum* until 9–12 weeks old. The study was approved by the Animal Experimentation Ethics Committee of Victoria University (AEC14/009) and conformed to the Australian National Code of Practice for the Care and Use of Animals for Scientific Purposes. The mice for each group in this study were randomly allocated.

### Muscle Dissection

Mice were fasted for 4 h before deep anaesthetization with 60 mg kg^−1^ intraperitoneal pentobarbital. Left and right EDL and soleus muscles were excised within 30 min of anesthesia. Isolated muscles were bathed in carbogenated Krebs–Henseleit buffer (KHB) (119 mM NaCl, 4.7 mM KCl, 2.5 mM CaCl_2_, 1.2 mM MgSO_4_, 1.2 mM KH_2_PO_4_, 25 mM NaHCO_3_, pH 7.4) and evenly divided into halves longitudinally. After muscle dissection, mice were euthanized *via* cervical dislocation under anesthesia.

### ucOC Stimulation

Muscles were evenly divided longitudinally into halves to improve the effusion of ucOC into muscle fiber *ex vivo*, similar to what has been performed in rat muscle in previous studies (16–18). The whole ucOC stimulation process is shown in Figure S1 in Supplementary Material. In experiments without the ERK inhibitor U0126, muscle samples were preincubated in 30°C baths containing carbogenated KHB buffer for 1 h. In experiments with U0126 (*N* = 5), after 30 min preincubation, muscle samples were exposed to the ERK inhibitor U0126 (1 µM) (Cell Signaling, MA, USA) or dimethyl sulfoxide (DMSO) vehicle (Sigma-Aldrich, MO, USA) for 30 min. Then, muscle samples were stimulated for 90 min with increasing doses [0 ng mL^−1^ (*N* = 6), 0.3 ng mL^−1^ (*N* = 10), 3 ng mL^−1^ (*N* = 10), 10 ng mL^−1^ (*N* = 14), or 30 ng mL^−1^ (*N* = 10)] of recombinant ucOC (Bachem, Bubendorf, Switzerland). These doses of ucOC were chosen because they are within the physiological range in mice (7, 31). In experiments without U0126, muscle halves from the same mouse were treated with KHB buffer control or ucOC. In experiments with U0126, muscle halves from the same mouse were treated with DMSO, DMSO with ucOC, U0126, and U0126 with ucOC, respectively.

### 2-Deoxyglucose Uptake Measurement and Sample Homogenization

The method to assess 2-Deoxy-D-glucose (2-DG) uptake has been described previously (14). Briefly, after the 90 min ucOC treatment, muscles were transferred to chambers containing KHB + 0.1% bovine serum albumin (Sigma-Aldrich) + 2 mM 2-Deoxy-d-[1,2-^3^H]-glucose (PerkinElmer, MA, USA) and 16 mM d-[1-^14^C] mannitol (PerkinElmer) with or without U0126/Vehicle or ucOC. After 10 min, muscles were rapidly rinsed with ice cold KHB buffer, then immediately frozen in liquid nitrogen. On the day of sample processing, muscle samples were lysed in ice-cold radioimmunoprecipitation assay (RIPA) buffer (60 µL RIPA for 1 mg sample) (Cell Signaling) with Inhibitor Cocktail (Cell Signaling) and 100 mM dithiothreitol (Sigma-Aldrich) using TissueLyser II (QIAGEN, Hilden, Germany) followed by gentle rocking at 4°C for 1 h. Half of the lysate was pipetted into vials with scintillation cocktail for scintillation counting (β-counter) with Tri-Carb 2910TR Liquid Scintillation Analyzer (PerkinElmer) and the other half was used in western blotting.

### Western Blotting

After muscle samples were homogenized using RIPA buffer, protein concentrations in the lysate were determined by Bio-Rad Protein Assay (Bio-Rad, CA, USA). Equal amounts of protein were subjected to electrophoresis on Criterion stain-free precast gels (10%; Bio-Rad) and then transferred electrophoretically using Trans-Blot Turbo Transfer System (Bio-Rad) onto a polyvinylidene fluoride membrane (Bio-Rad). Then, a stain-free blot image was taken using ChemiDoc Imaging System (Bio-Rad) for total protein measurement in each sample lane. Immunoblotting was performed at optimum conditions for each antibody. Bands were identified using ChemiDoc Imaging System, using SuperSignal West Femto Maximum Sensitivity Substrate (Thermo, MA, USA). Band densities of both stain-free blot and immunoblotting were measured using Image Lab Software (Bio-Rad). Values of immunoblotting bands were normalized using total protein values. p-ERK (Thr202/Tyr204), ERK, p-AMPKα (Thr172), AMPKα, p-mTOR (Ser2481), mTOR, p-AKT (Ser473), AKT, p-AS160 (Thr642), AS160, and p-PKCδ/θ (Ser643/676) antibodies were purchased from Cell Signaling.

Two data points for AMPKα phosphorylation assessment were excluded due to western blot imaging artifacts. However, their exclusion did not alter the statistical outcome, interpretation, or conclusions of the results.

### Statistical Analysis

Fold-changes for western blotting data were calculated by normalization to control groups within the same animals. 3 ng mL^−1^ group and 30 ng mL^−1^ group were chosen for western blotting and correlation analysis as representatives of low and high doses of ucOC.

Paired *t*-tests were used to analyze the effects of ucOC, for each individual concentration, on muscle glucose uptake, protein phosphorylation, protein abundance, and phospho/total ratio compared to paired control samples. This paired comparison was used to exclude individual variances.

To analyze the dose–response effects of ucOC on muscle glucose uptake, basal glucose uptake data from all groups were combined, then one-way ANOVA with Tukey *post hoc* test was applied.

Spearman’s correlation was performed between the variables from 30 ng mL^−1^ ucOC treatment group. Rule of thumb for interpreting the size of a correlation coefficient will be applied to measure the strength of correlation between two variables (32). According to standard practice thresholds, the *r* ranges for negligible positive, low positive, moderate positive, high positive, and very high positive correlations are defined as 0.00 < *r* < 0.30, 0.30 ≤ *r* < 0.50, 0.50 ≤ *r* < 0.70, 0.70 ≤ *r* < 0.90, and 0.90 ≤ *r* ≤ 1.00, respectively.

All figures and analyses were performed using GraphPad 6 (GraphPad Software, La Jolla, CA, USA).

All data are reported as mean ± SEM.

## Results

### ucOC Increased Glucose Uptake in both EDL and Soleus Muscles

**Figure 1:**
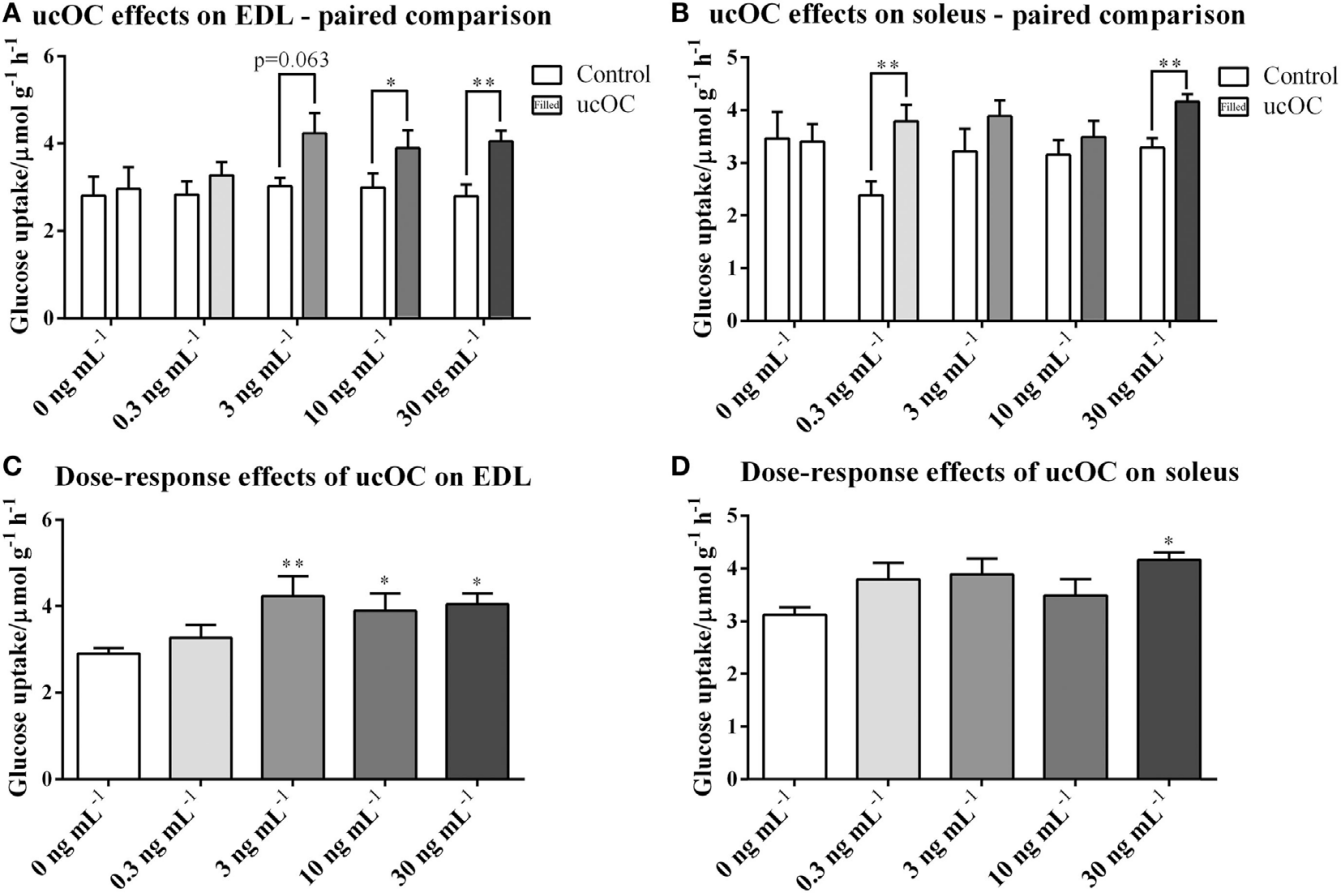
Undercarboxylated osteocalcin (ucOC) effects on and insulin-stimulated glucose uptake in extensor digitorum longus (EDL) and soleus. **(A,B)** glucose uptake of EDL and soleus muscle samples treated with Krebs–Henseleit buffer control and ucOC [0 ng mL^−1^ (mice *N* = 6), 0.3 ng mL^−1^ (mice *N* = 10), 3 ng mL^−1^ (mice *N* = 10), 10 ng mL^−1^ (mice *N* = 14), or 30 ng mL^−1^ (mice *N* = 10)] was detected. *P ≤ 0.05 and **P ≤ 0.01 between paired samples (paired *t*-test); **(C,D)** glucose uptake of EDL and soleus samples was analyzed for dose-response effect of ucOC with combined basal levels. Samples sizes for groups treated with 0, 0.3, 3, 10, or 30 ng mL^−1^ ucOC are 56, 10, 10, 14, or 10, respectively. *P ≤ 0.05 and **P ≤ 0.01 in Tukey’s *post hoc* test (compared with 0 ng mL^−1^ ucOC samples) of one-way ANOVA analysis.

Compared with paired controls, muscle glucose uptake was significantly higher following the treatment of ucOC at doses of 10 ng mL^−1^ (*P* < 0.05) and 30 ng mL^−1^ (*P* < 0.01) in EDL, and 0.3 ng mL^−1^ (*P* < 0.01) and 30 ng mL^−1^ (*P* < 0.01) in soleus (Figures 1A,B). When data were analyzed for ucOC dose–response effects, ucOC significantly enhanced glucose uptake at doses equal or larger than 3 ng mL^−1^ in EDL and at a dose of 30 ng mL^−1^ in soleus (Figures 1C,D; *P* < 0.05, ANOVA *P* < 0.01), from 2.91 to 4.32 μmol g^−1^ h^−1^ and from 3.12 to 4.16 μmol g^−1^ h^−1^, respectively.

### ucOC Stimulated the Phosphorylation of mTOR, AKT, and AS160

**Figure 2:**
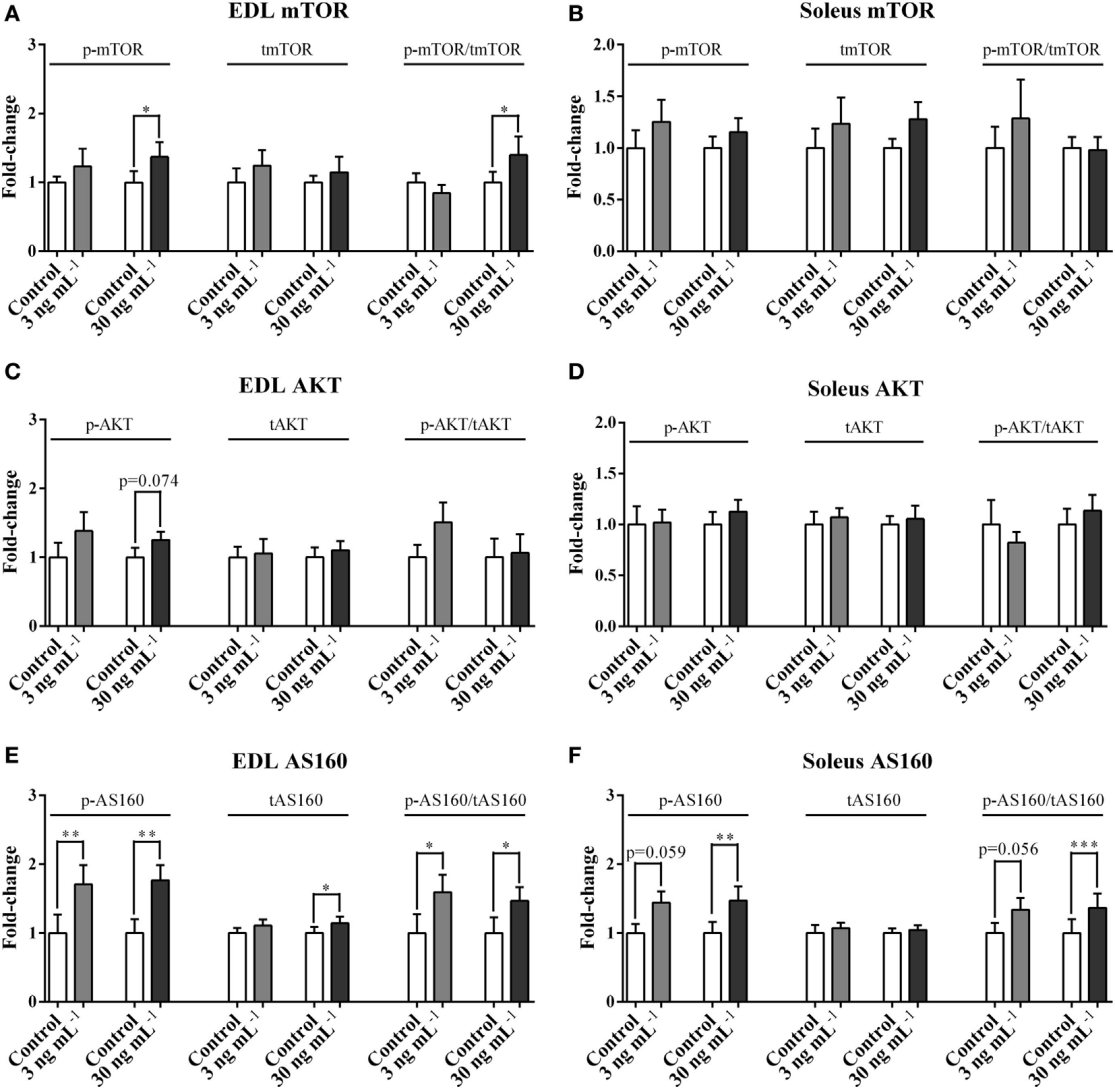
Undercarboxylated osteocalcin (ucOC) effects on the phosphorylation of mTOR, AKT, and AS160. The phosphorylation levels, total expression levels, and phospho/total ratio levels of mTOR **(A,B)**, AKT **(C,D)**, and AS160 **(E,F)** of Extensor digitorum longus (EDL) and soleus samples treated with Krebs–Henseleit buffer control and ucOC (3 and 30 ng mL^−1^, *N* = 10 for each dose) were examined. *P ≤ 0.05, **P ≤ 0.01, and ***P ≤ 0.001 paired samples from the same animal (*t*-test).

In EDL, ucOC treatment at 30 ng mL^−1^ significantly increased p-mTOR (1.37-fold, *P* < 0.05, Figure 2A) and p-mTOR/tmTOR ratio (1.40-fold, *P* < 0.05, Figure 2A), and only tended to increase p-AKT (1.25-fold, *P* = 0.074, Figure 2C) but not p-AKT/tAKT ratio. Neither of these signaling molecules was affected in the soleus (Figures 2B,D). In both EDL and soleus, both p-AS160 and p-AS160/tAS160 ratio were considerably elevated 1.4-fold to 1.8-fold following ucOC treatments at 3 ng mL^−1^ (*P* < 0.05 and *P* < 0.05, Figure 2E; *P* = 0.059 and *P* = 0.056, Figure 2F) and 30 ng mL^−1^ (*P* < 0.01 and *P* < 0.05, Figure 2E; *P* < 0.01 and *P* < 0.001, Figure 2F). Total AS160 expression was also increased by 30 ng mL^−1^ ucOC in EDL (1.13-fold, *P* < 0.05, Figure 2E). Blots of phosphorylated proteins and total expression of proteins are shown as Figure S2 in Supplementary Material.

### ucOC Stimulated the Phosphorylation of ERK but Not AMPK

**Figure 3:**
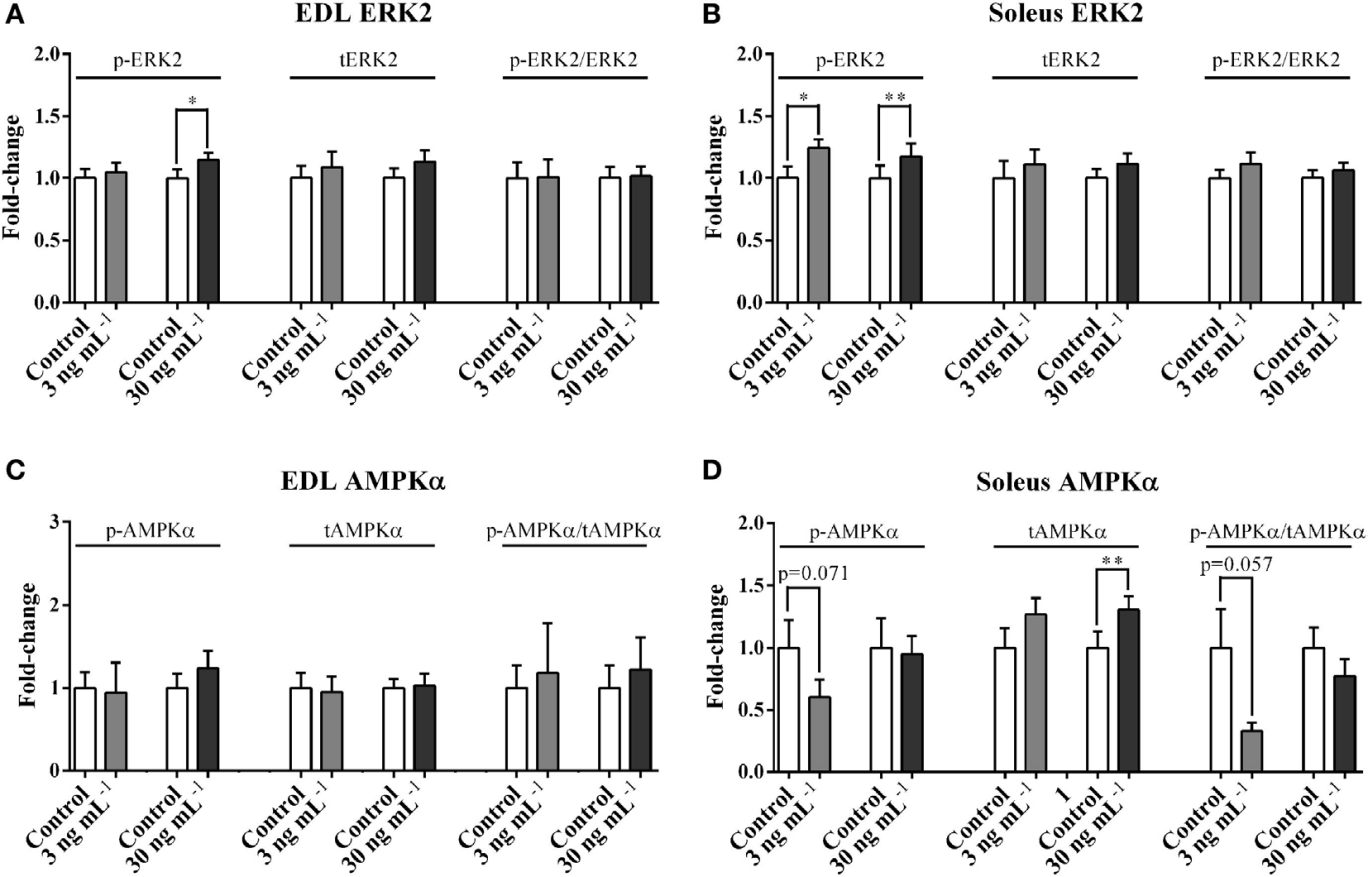
Undercarboxylated osteocalcin (ucOC) effects on the phosphorylation of ERK2 and AMPKα. The phosphorylation levels, total expression levels, and phospho/total ratio levels of ERK2 **(A,B)** and AMPKα **(C,D)** of Extensor digitorum longus (EDL) and soleus samples treated with Krebs–Henseleit buffer control and ucOC (3 and 30 ng mL^−1^, *N* = 9–10 for each dose) were examined. *P ≤ 0.05 and **P ≤ 0.01 paired samples from the same animal (*t*-test).

p-ERK2, but not p-ERK2/tERK2 ratio, was increased by the treatment of 30 ng mL^−1^ ucOC (1.14-fold, *P* < 0.05, Figure 3A) in EDL, and by both 3 and 30 ng mL^−1^ of ucOC in soleus (1.24-fold and 1.17-fold, *P* < 0.05 and *P* < 0.01, Figure 3B). ucOC at 3 or 30 ng mL^−1^ had limited effects on AMPKα phosphorylation in both EDL and soleus (Figures 3C,D). However, soleus total AMPKα levels were increased by the treatment of 30 ng mL^−1^ ucOC (1.30-fold, *P* < 0.01, Figure 3D). Blots of phosphorylated proteins and total expression of proteins are shown as Figure S3 in Supplementary Material.

Treatment with 30 ng mL^−1^ ucOC had limited effects on phosphorylated protein kinase C δ/θ (PKCδ/θ) in both EDL and soleus muscles (Figure S4 in Supplementary Material).

### The Phosphorylation Levels of ERK2 Correlated with the Phosphorylation Levels of AKT and AS160

**Figure 4:**
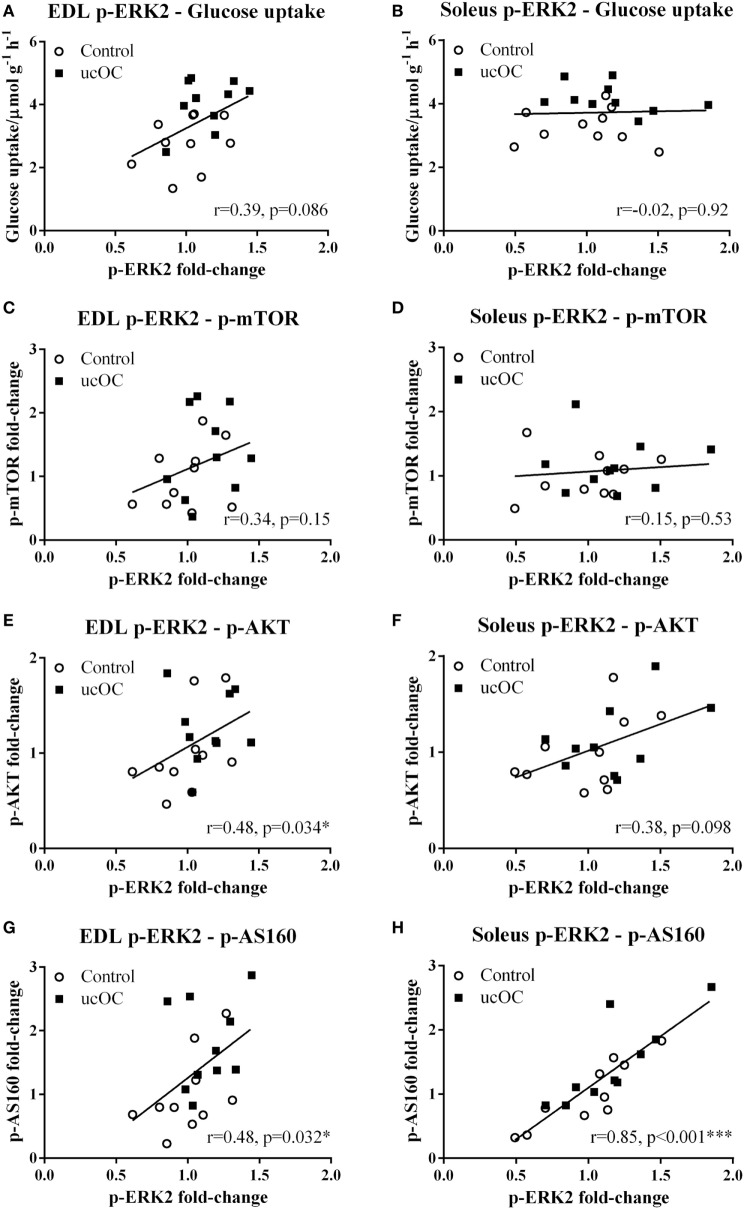
The correlations between the levels of p-ERK2 and the levels of glucose uptake, p-mTOR, p-AKT, and p-AS160. In extensor digitorum longus (EDL) and soleus samples, the correlations between the levels p-ERK2 fold-change and the levels of glucose uptake **(A,B)**, p-mTOR fold-change **(C,D)**, p-AKT fold-change **(E,F)**, and p-AS160 fold-change **(G,H)** were analyzed among samples from 30 ng mL^−1^ undercarboxylated osteocalcin (ucOC) treatment group; *P ≤ 0.05 and ***P ≤ 0.001.

p-ERK2 levels were not associated with glucose uptake levels (Figures 4A,B) or p-mTOR levels (Figures 4C,D), in either EDL or soleus. Higher levels of p-ERK2 were associated with higher levels of p-AKT in EDL (*P* < 0.05, Figure 4E) with a low positive correlation (*r* = 0.48), but not in soleus (Figure 4F). In both EDL (*P* < 0.05, Figure 4G) and soleus (*P* < 0.001, Figure 4H), higher p-ERK2 levels were associated with higher levels of p-AS160, with a low positive correlation (*r* = 0.48) and a high positive correlation (*r* = 0.85), respectively.

p-AMPKα levels were not associated with glucose uptake or any signaling protein phosphorylation levels in either muscle type (data not shown).

### The Prevention of ucOC-Induced ERK Phosphorylation Had Limited Effect on ucOC-Stimulated Muscle Glucose Uptake

**Figure 5:**
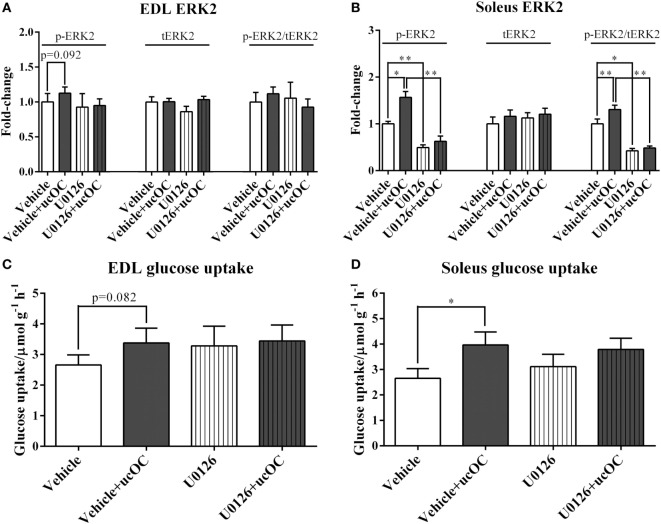
The effects of the removal of p-ERK2 enhancement by U0126 on undercarboxylated osteocalcin (ucOC)-stimulated muscle glucose uptake. **(A,B)** The phosphorylation levels, total expression levels, and phospho/total ratio levels of ERK2 in samples treated with dimethyl sulfoxide (DMSO) vehicle, vehicle plus 30 ng mL^−1^ ucOC, U0126 (1 μM), and U0126 plus ucOC were assessed in Extensor digitorum longus (EDL) and soleus muscles; **(C,D)** the glucose uptake of samples treated with DMSO vehicle, vehicle plus ucOC (30 ng mL^−1^), U0126 (1 μM), or U0126 plus ucOC were examined in EDL and soleus muscles. *P ≤ 0.05 and **P ≤ 0.01 between paired samples (*t*-test).

Preincubation with 1 µM U0126 blocked ucOC (30 ng mL^−1^)-induced increases in ERK2 phosphorylation in both EDL and soleus (Figures 5A,B). However, the addition of inhibitor did not significantly affect ucOC-stimulated muscle glucose uptake (Figures 5C,D).

### The Removal of ucOC-Induced ERK Phosphorylation Prevents ucOC-Stimulated AKT Phosphorylation in EDL, but Has Limited Effect on the Phosphorylation of mTOR and AS160 in Both Muscle Types

**Figure 6:**
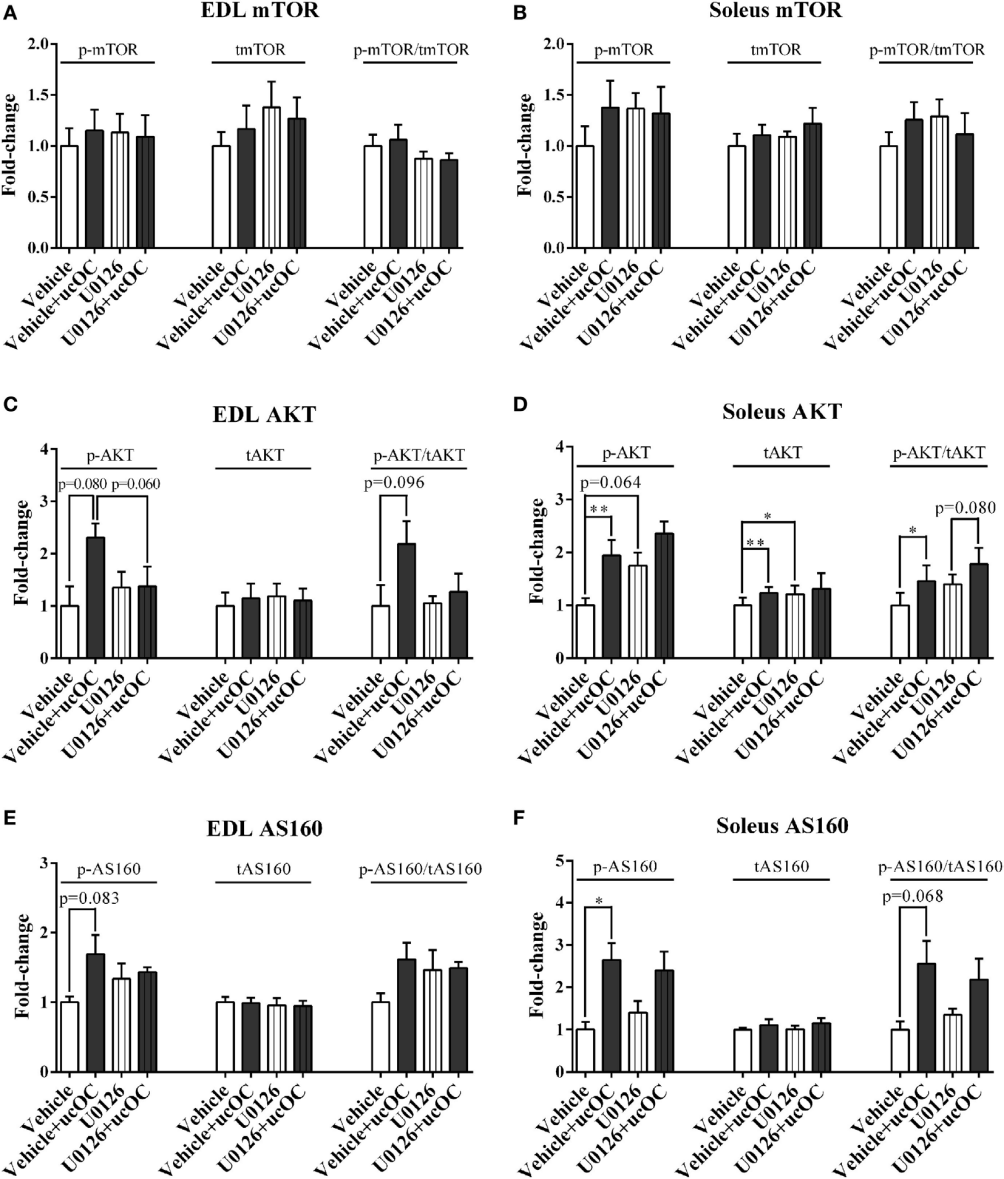
The effects of the removal of p-ERK2 enhancement by U0126 on undercarboxylated osteocalcin (ucOC)-stimulated p-mTOR, p-AKT, and p-AS160. The phosphorylation levels, total expression levels, and phospho/total ratio levels of mTOR **(A,B)**, AKT **(C,D)**, and AS160 **(E,F)** of samples treated with dimethyl sulfoxide vehicle, vehicle plus 30 ng mL^−1^ ucOC, 1 μM U0126, and U0126 plus ucOC were assessed in extensor digitorum longus (EDL) and soleus muscles (*N* = 5). *P ≤ 0.05 and **P ≤ 0.01 between paired samples from the same animal (*t*-test).

U0126 (1 µM) had limited effect on p-mTOR following ucOC treatment in either EDL or soleus (Figures 6A,B). However, it somewhat prevented the ucOC-mediated AKT activation in EDL with a change close to significant observed in phosphorylation levels (*P* = 0.06), but not in soleus (Figures 6C,D). Although AS160 phosphorylation shared similar patterns of modulation with those of AKT following the treatments, ucOC-stimulated AS160 phosphorylation levels were only marginally decreased by U0126 addition in both muscle types (*P* > 0.1; Figures 6E,F).

## Discussion

We report that physiological levels of ucOC *per se* increased muscle glucose uptake *ex vivo* in both EDL (glycolytic muscle) and soleus (oxidative muscle) muscles. Furthermore, ucOC increased the phosphorylation of ERK2, mTOR, and AS160 in EDL and enhanced the phosphorylation of ERK2 and AS160 in soleus muscle. It appears that ERK phosphorylation was not directly involved in ucOC-stimulated glucose uptake and AS160 phosphorylation in both muscle types.

We, and others, have previously reported that ucOC had no significant effect on resting EDL muscle glucose uptake, indicating that ucOC *per se* probably only upregulates muscle glucose metabolism in oxidative muscle fibers (14, 15). However, since the expression of GPRC6A, which is reported as the plausible receptor of ucOC, has been found in both muscle types (14, 15), we suggested that the results of these studies were affected by a potential methodological limitation that the usage of intact whole muscles may prevent adequate ucOC exposure to all muscle fibers. *In vivo*, muscle fibers are closely fed by capillaries that penetrate the epimysium and bifurcate throughout the muscle, primarily within perimysium (33). Since both epimysium and perimysium belong to robust collagenous connective tissue networks, without the help of blood vessels, ucOC in external solution may have limited direct contact with fibers of intact muscles during *ex vivo* incubation. By utilizing the method of splitting muscles longitudinally into halves (16–18), in order to increase the ucOC saturation during treatment, we report that ucOC can increase muscle glucose uptake in the absence of insulin in both glycolytic (EDL) and oxidative (soleus) muscles, suggesting the effect of ucOC on skeletal muscle glucose uptake is likely universal rather than muscle-type specific. It also seems that, compared with EDL, higher doses of ucOC are required for observing this effect on the glucose uptake of soleus (Figures 1C,D). Although there was a significant increase in soleus treated with 0.3 ng mL^−1^ ucOC using paired comparison method (Figure 1B), this increase could not be observed when data were analyzed using one-way ANOVA. Thus, it was likely that this increase was merely resulted from an abnormally low control levels in that specific group. Since skeletal muscle is the major site of glucose disposal and utilization in the postprandial state (9), these findings implicate ucOC as a possible therapeutic agent to improve muscle glucose transport even without insulin.

However, it should be noted that even though we introduced muscle splits in this study to enhance the interaction between ucOC and interior muscle myotubes beneath muscle surface, some limitations, which might result in enhanced biological variations, such as different ucOC saturation percentages due to different muscle dimensions, and different basal glucose uptake levels of different individuals, still cannot be ruled out. Therefore, future studies should explore the effect of ucOC in primary myotubes from animals and human, to avoid these limitations.

We report that ucOC treatment activated the mTORC2-AKT-AS160 signaling cascade in skeletal muscle, in a muscle type-specific manner. Importantly, ucOC elicited significant increases in AS160 phosphorylation (Thr642) despite relatively modest increases in AKT phosphorylation (Ser473) (*P* = 0.074). Therefore, ucOC may enhance AS160 phosphorylation *via* AKT independent mechanisms, which may be the major mechanisms underlying the ucOC-induced enhancement of glucose uptake and AS160 phosphorylation. Indeed, several other signaling proteins are also able to increase AS160 phosphorylation, including AMPK and conventional/novel (c/n) protein kinase C (PKC) (34).

Along with ERK, previous studies have suggested that AMPK is a potential downstream target of the ucOC signaling in skeletal muscle (13, 15). Our data shows that following 30 ng mL^−1^ ucOC treatment, there is a significant increase in phosphorylated ERK2 (Thr202/Yyr204) in both EDL and soleus muscles. However, there was limited change in AMPKα phosphorylation at Thr172, a phosphorylation site that has widely been reported as an indicator of AMPK activity (35–37). Similarly, it has been reported that AMPKα Thr172 phosphorylation was not increased following ucOC treatment in C2C12 cells (13). A recent paper reported that intraperitoneal injection of osteocalcin increased AMPK phosphorylation in mouse muscles during exercise (15). Given that AMPK Thr172 phosphorylation is increased in skeletal muscle after acute exercise alone, without ucOC (38, 39), it is possible that ucOC treatment merely has an additive effect on exercise-enhanced phosphorylation of AMPK, but is unable to increase its phosphorylation level *per se*. However, it should be noted that until now no studies have shown ucOC-induced change of AMPK phosphorylation over the course of time. Thus, a transient increase of AMPK phosphorylation after ucOC treatment still cannot be ruled out.

We neither observed any significant increases in the phosphorylation in PKCδ/θ, two important members in novel PKC family, in both muscle types (Figure S4 in Supplementary Material). This finding suggests a limited role of PKCδ/θ in the modulation of muscle glucose uptake by ucOC. However, the involvement of other types of PKC in the mechanisms behind this ucOC effect is still possible, which warrants further investigation.

We reported that p-AKT in EDL and p-ERK2 in both EDL and soleus were enhanced following ucOC treatment with no significant changes in phospho/total ratio (Figures 2C and 3A,B). This discrepancy may be attributed to an ucOC-induced modest increase in total protein expression (Figures 2E and 3D), in addition to its effect on protein phosphorylation. Consistent with our finding, protein synthesis has recently been reported to increase in mouse myotubes following 1–2 h of ucOC treatment (40, 41). It is possible that ucOC regulates kinase activity by both enhancing protein phosphorylation, and, to a lesser extent, increasing protein abundance.

We report a low positive correlation between p-ERK2 levels and p-AKT levels as well as a high positive correlation between p-ERK2 levels and p-AS160 levels in EDL, which was consistent with our previous findings showing that lower p-ERK levels were associated with lower p-AKT levels in rat EDL muscle (30). In soleus, p-ERK2 levels were associated with p-AS160 levels, with a low positive correlation. As such, we investigated whether the removal of ucOC-mediated ERK phosphorylation leads to the suppression of the effect of ucOC on skeletal muscles. Pretreatment with 1 µM U0126 blocked ucOC-stimulated increases in ERK phosphorylation in both muscle types. In EDL, it seems that the inhibition of p-ERK2 blocked ucOC-stimulated AKT phosphorylation (*P* = 0.06). Consistently, a previous finding also suggested that partial ERK inhibition dampened ucOC-stimulated AKT phosphorylation in C2C12 myotubes (13). In contrast, in response to the loss of ucOC-induced ERK phosphorylation, ucOC-stimulated glucose uptake and AS160 phosphorylation was not compromised. Similarly, in soleus, the inhibition of p-ERK2 had limited effects on ucOC-stimulated glucose uptake and the phosphorylation of signaling proteins. These findings suggest that mechanisms underlying ucOC stimulation in skeletal muscle are probably muscle type-specific, but converging at AS160 phosphorylation, both resulting in the enhancement of glucose uptake. The ucOC-stimulated mTOR phosphorylation was also not modulated by U0126 pretreatment in both muscle types. Thus, whether ERK signaling modulates AKT phosphorylation through mTORC2 needs further investigations.

The administration of U0126 as an ERK inhibitor has limitations. One limitation derives from the influence of vehicle DMSO, which is widely used as the solvent for U0126. DMSO has been shown to exert some impact on skeletal muscle, such as depressing muscle contractility and accelerating muscle injury (42, 43). In our study, by comparing the results shown in Figure 2 with those in Figure 6, it is suggested that even the presence of a low concentration of DMSO (0.1%) may have slightly altered the ucOC effect on the phosphorylation of mTOR in EDL and AKT in soleus. However, since DMSO was universally added to all samples in experiments involving the prevention of ucOC-induced ERK phosphorylation, the conclusions drawn from comparisons between these samples are unlikely to be affected by DMSO addition. The other limitation of U0126 administration in this study is the capability of U0126 to enhance glucose uptake and AKT phosphorylation by itself (44–46). It has been suggested that this effect is due to the elevation of AMPK activity that is independent of ERK inhibition (47). In the current study, the application of low dose (1 μM) of U0126 had a limited effect on AMPK phosphorylation in both muscle types (Figure S6 in Supplementary Material), and glucose uptake and phosphorylation of most other signaling proteins were also minimally or not at all affected. Nevertheless, other inhibitors or methodologies for ERK inhibition should be investigated in future studies to confirm the involvement of ERK in the regulation of AKT phosphorylation.

In conclusion, ucOC increases glucose uptake in both glycolytic and oxidative muscles in the absence of insulin, *via* mechanisms involving enhanced AS160 phosphorylation. Therefore, ucOC should be considered as a potential agent to improve muscle glucose uptake in insulin resistant conditions including type 2 diabetes.

## Ethics Statement

The study was approved by the Animal Experimentation Ethics Committee of Victoria University (AEC14/009) and conformed to the Australian National Code of Practice for the Care and Use of Animals for Scientific Purposes.

## Author Contributions

XL, XZ, TB-S, and IL design the study. XL, LP, and EM performed experiments and data collection. XL and LP did the data analysis. XL wrote the paper. XL, LP, XZ, AH, GM, TB-S, and IL substantially contributed to manuscript revision and approved the final version.

## Conflict of Interest Statement

The authors declare that the research was conducted in the absence of any commercial or financial relationships that could be construed as a potential conflict of interest.

## Supplementary Material

Figure S1 Flow-chart of the protocol used in this study.

Figure S2 Blots of all samples in western blot analysis for p-mTOR, p-AKT, and p-AS160. Blots of p-mTOR at Ser2481 in Extensor digitorum longus (EDL) **(A)** and soleus **(B)** samples, p-AKT at Ser473 in EDL **(C)** and soleus **(D)** samples, and p-AS160 at Thr642 in EDL **(E)** and soleus **(F)** samples from 3 ng mL^−1^ (*N* = 10) and 30 ng mL^−1^ (*N* = 10) groups are exhibited.

Figure S3 Blots of all samples in western blot analysis for p-ERK2 and p-AMPKα. Blots of p-ERK2 at Thr202/Tyr204 in Extensor digitorum longus (EDL) **(A)** and soleus **(B)** samples as well as p-AMPKα at Thr172 in EDL **(C)** and soleus **(D)** samples from 3 ng mL^−1^ (*N* = 9–10) and 30 ng mL^−1^ (*N* = 9–10) groups are exhibited.

Figure S4 The phosphorylation levels of protein kinase C (PKC)δ/θ in Extensor digitorum longus and soleus samples treated with Krebs–Henseleit buffer buffer control and 30 ng mL^−1^ ucOC were examined (*N* = 10).

Figure S5 Blots of all samples in western blot analysis for p-ERK2, p-mTOR, p-AKT, and p-AS160 in ERK inhibition experiments. Blots of p-ERK2 at Thr202/Tyr204 in Extensor digitorum longus (EDL) **(A)** and soleus **(B)** samples, p-mTOR at Ser2481 in EDL **(C)** and soleus **(D)** samples, p-AKT at Ser473 in EDL **(E)** and soleus **(F)** samples, and p-AS160 at Thr642 in EDL **(G)** and soleus **(H)** samples in ERK inhibition experiments are exhibited (*N* = 5).

Figure S6 The phosphorylation levels of AMPKα at Thr172 in Extensor digitorum longus (EDL) and soleus samples treated with treated with dimethyl sulfoxide vehicle, vehicle plus 30 ng mL^−1^ ucOC, 1 μM U0126, and U0126 plus ucOC were examined (*N* = 5).
