# Supplementary figures and images for: Recombinant Uncarboxylated Osteocalcin Per Se Enhances Mouse Skeletal Muscle Glucose Uptake in both Extensor Digitorum Longus and Soleus Muscles
Source: Front Endocrinol (Lausanne). 2017 Nov 22;8:330. doi: 10.3389/fendo.2017.00330 (PMC5698688; doi:10.3389/fendo.2017.00330)

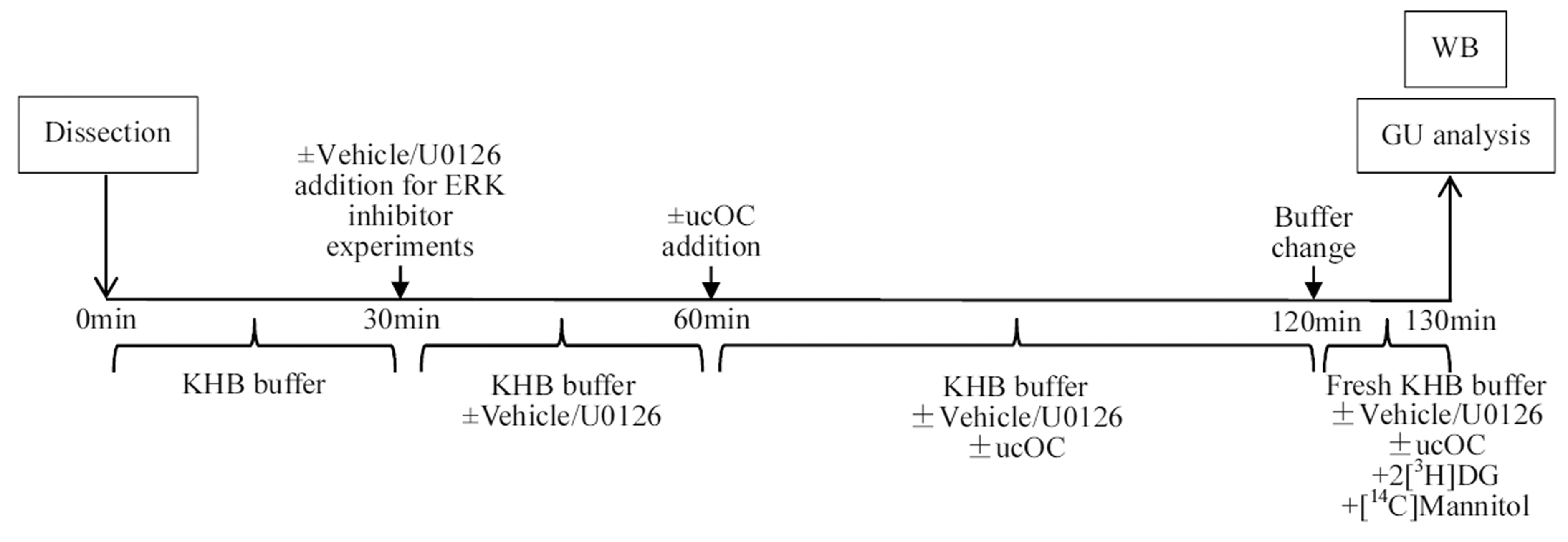

Supplement: Figure S1 — Flow-chart of the protocol used in this study. [file Image_1.tif]

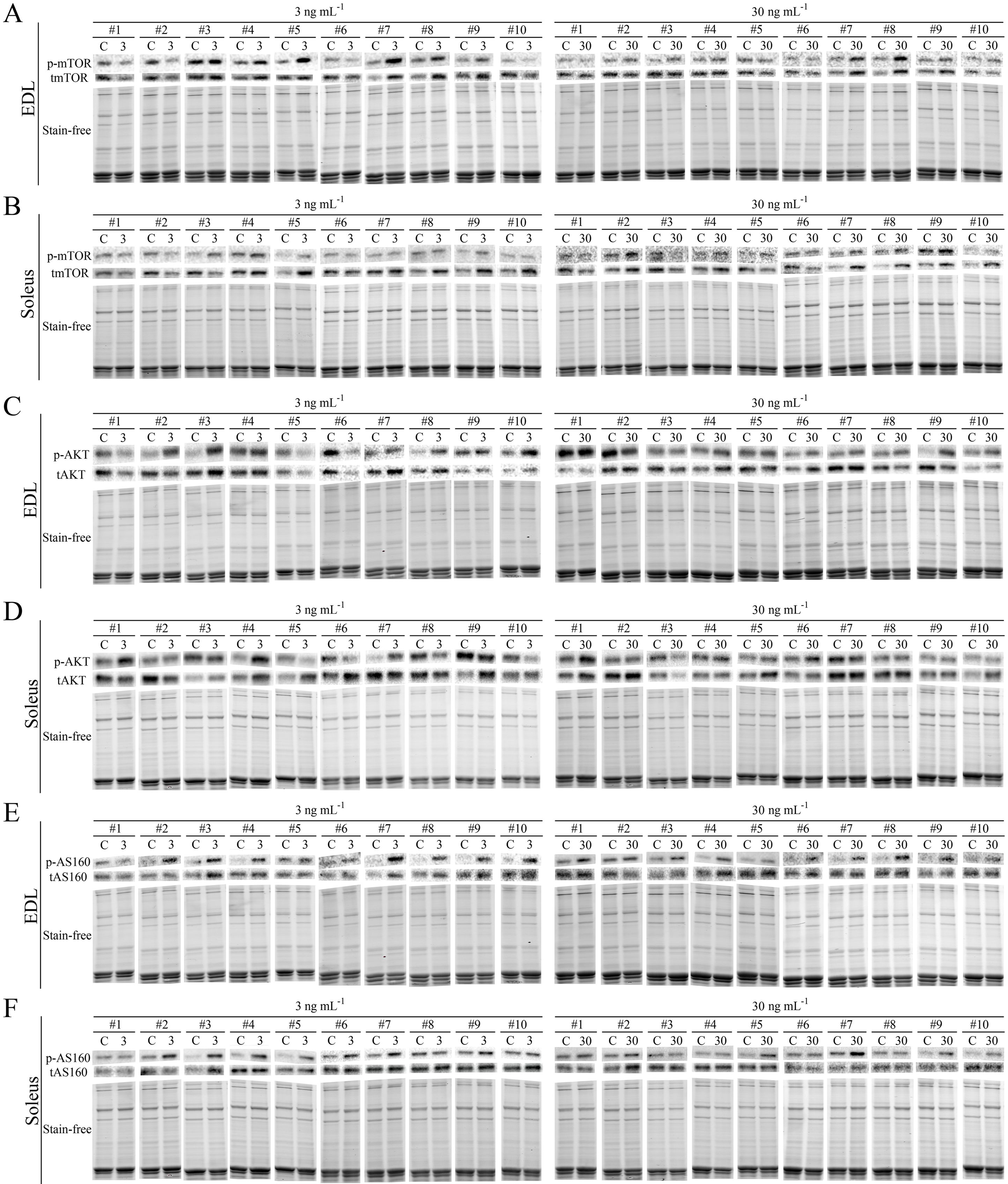

Supplement: Figure S2 — Blots of all samples in western blot analysis for p-mTOR, p-AKT, and p-AS160. Blots of p-mTOR at Ser2481 in Extensor digitorum longus (EDL) (A) and soleus (B) samples, p-AKT at Ser473 in EDL (C) and soleus (D) samples, and p-AS160 at Thr642 in EDL (E) and soleus (F) samples from 3 ng mL−1 (N = 10) and 30 ng mL−1 (N = 10) groups are exhibited. [file Image_2.tif]

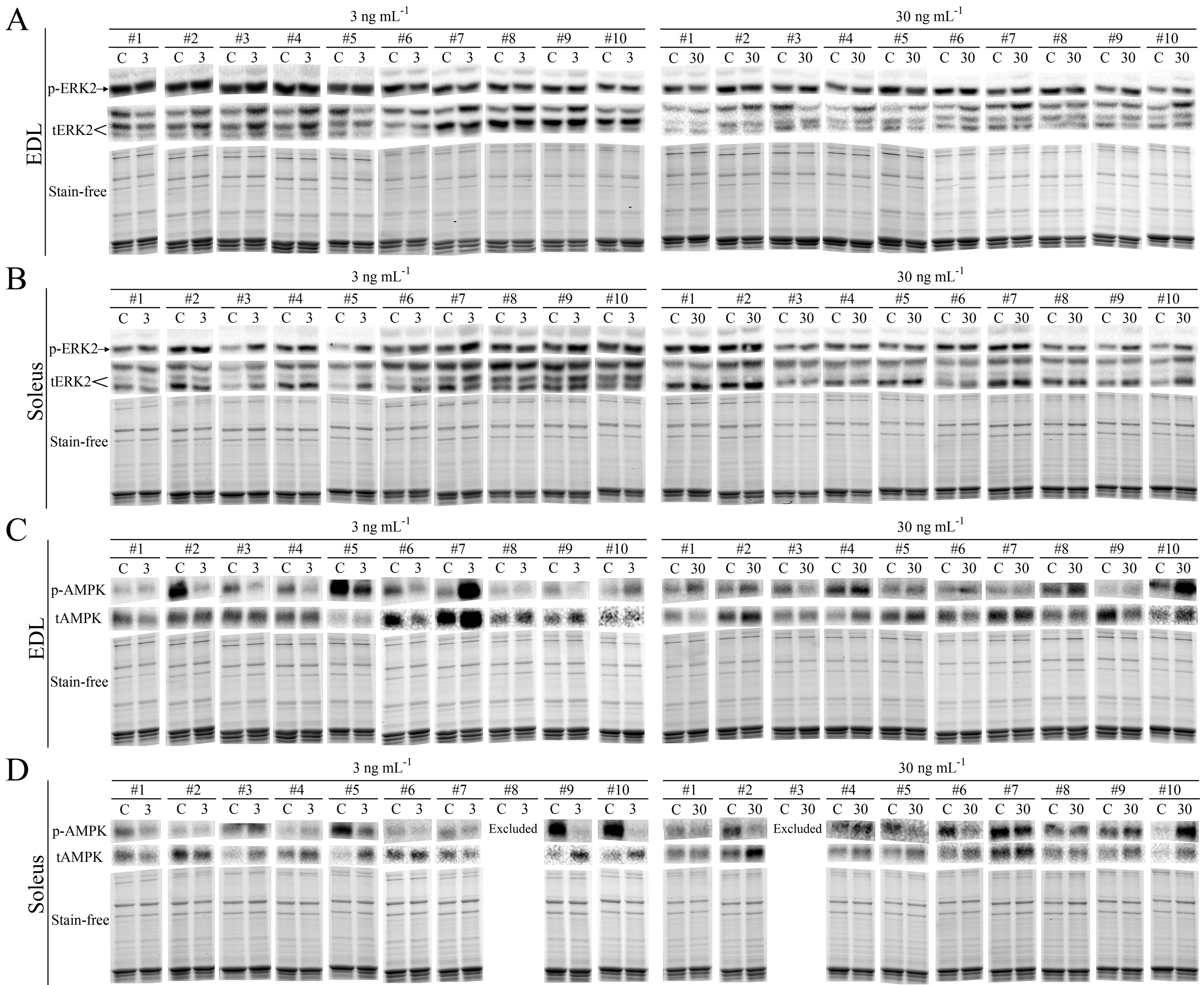

Supplement: Figure S3 — Blots of all samples in western blot analysis for p-ERK2 and p-AMPKα. Blots of p-ERK2 at Thr202/Tyr204 in Extensor digitorum longus (EDL) (A) and soleus (B) samples as well as p-AMPKα at Thr172 in EDL (C) and soleus (D) samples from 3 ng mL−1 (N = 9–10) and 30 ng mL−1 (N = 9–10) groups are exhibited. [file Image_3.tif]

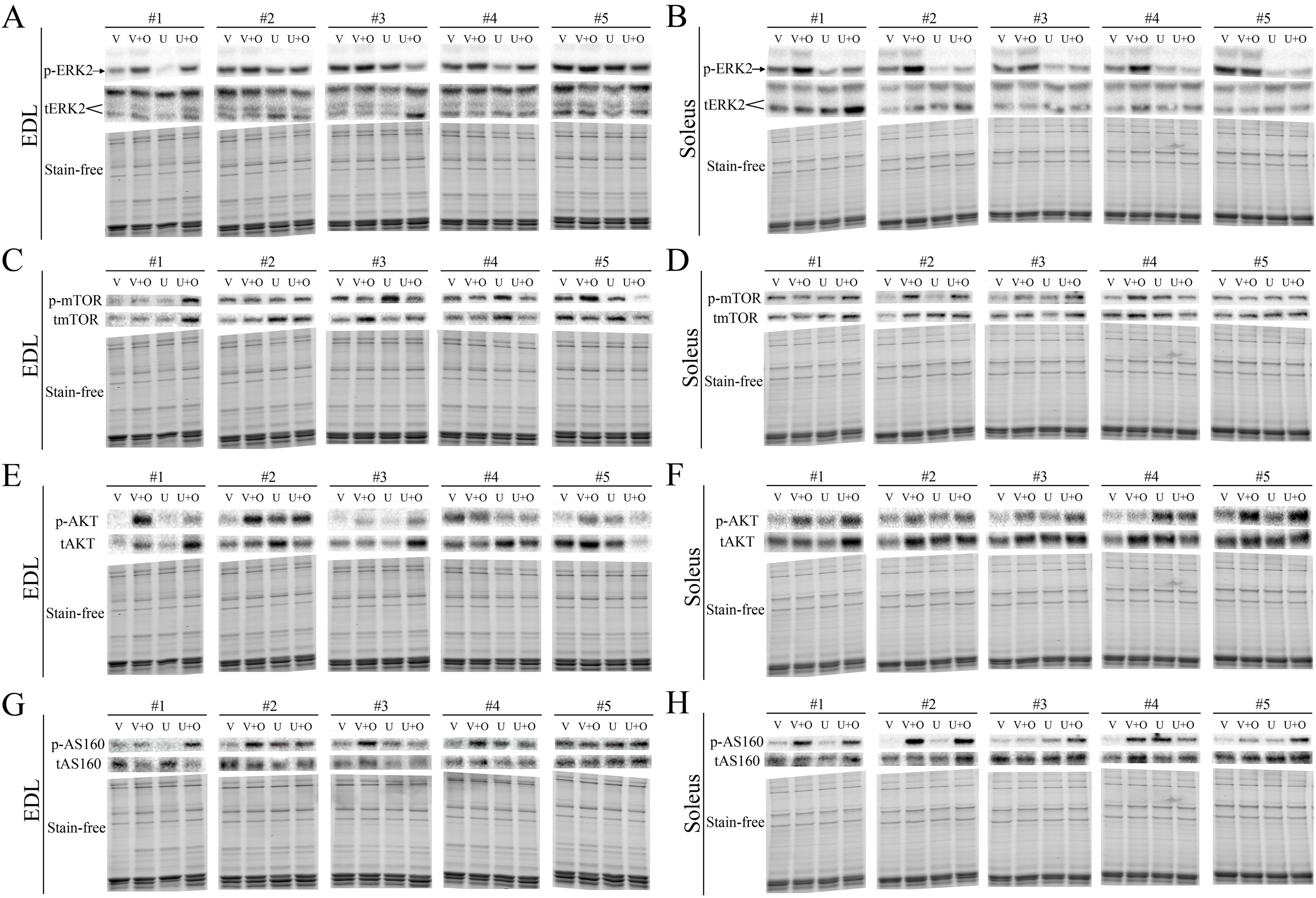

Supplement: Figure S5 — Blots of all samples in western blot analysis for p-ERK2, p-mTOR, p-AKT, and p-AS160 in ERK inhibition experiments. Blots of p-ERK2 at Thr202/Tyr204 in Extensor digitorum longus (EDL) (A) and soleus (B) samples, p-mTOR at Ser2481 in EDL (C) and soleus (D) samples, p-AKT at Ser473 in EDL (E) and soleus (F) samples, and p-AS160 at Thr642 in EDL (G) and soleus (H) samples in ERK inhibition experiments are exhibited (N = 5). [file Image_5.tif]

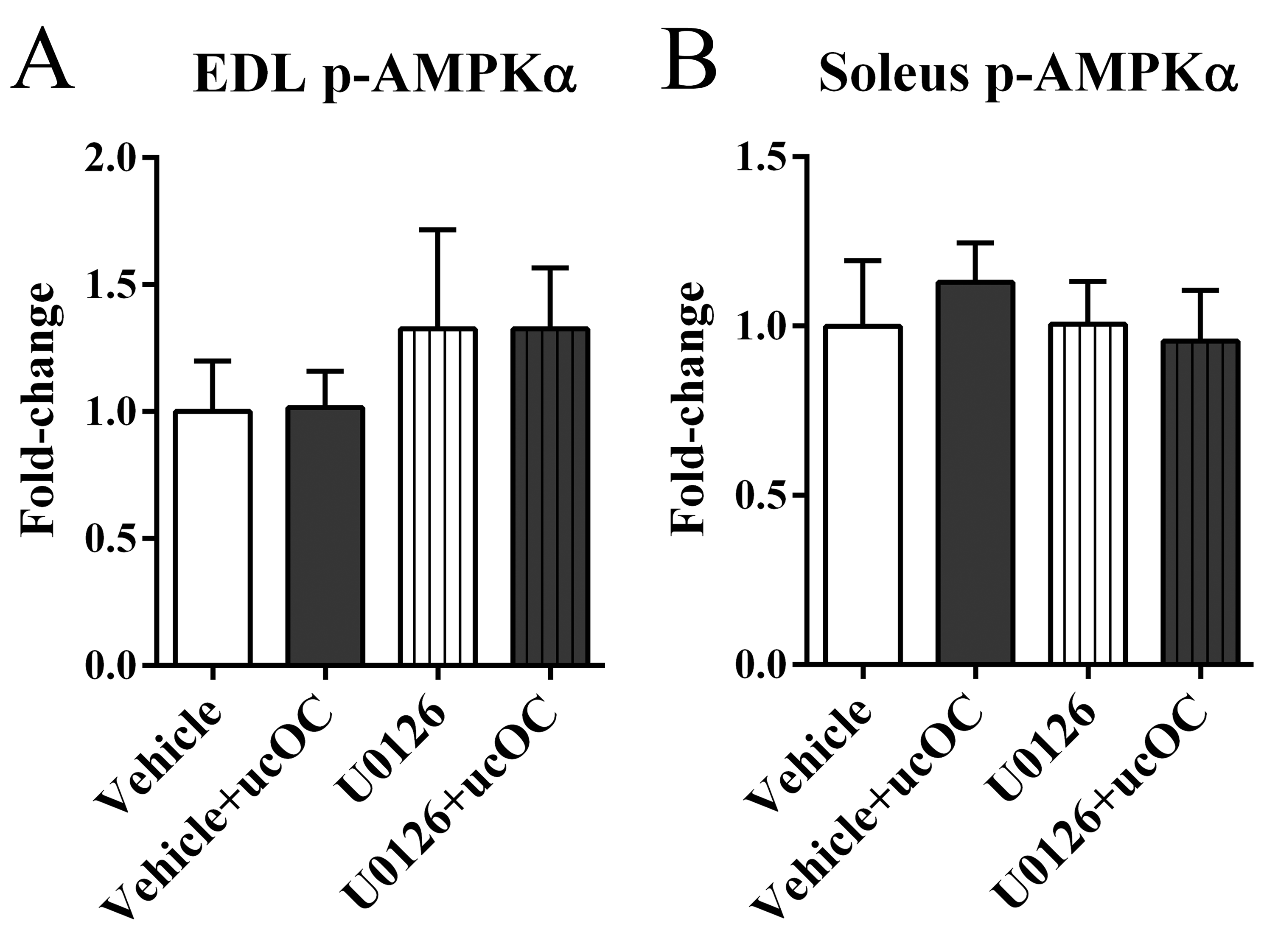

Supplement: Figure S6 — The phosphorylation levels of AMPKα at Thr172 in Extensor digitorum longus (EDL) and soleus samples treated with treated with dimethyl sulfoxide vehicle, vehicle plus 30 ng mL−1 ucOC, 1 μM U0126, and U0126 plus ucOC were examined (N = 5). [file Image_6.tif]
